# Reprogramming the adjuvant properties of aluminum oxyhydroxide with nanoparticle technology

**DOI:** 10.1038/s41541-018-0094-0

**Published:** 2019-01-03

**Authors:** Mark T. Orr, Amit P. Khandhar, Emilie Seydoux, Hong Liang, Emily Gage, Traci Mikasa, Elyse L. Beebe, Nicholas D. Rintala, Karin H. Persson, Anwar Ahniyaz, Darrick Carter, Steven G. Reed, Christopher B. Fox

**Affiliations:** 1 Infectious Disease Research Institute, 1616 Eastlake Avenue East, Suite 400, Seattle, WA 98102 USA; 2 Department of Global Health, University of Washington, 1510 San Juan Road, Seattle, WA 98195 USA; 3 Unit of Surface, Process and Formulation, Division of Bioscience and Materials, RISE Research Institutes of Sweden, Drottning Kristinas Väg 45, Box 5607, SE 11486 Stockholm, Sweden

## Abstract

Aluminum salts, developed almost a century ago, remain the most commonly used adjuvant for licensed human vaccines. Compared to more recently developed vaccine adjuvants, aluminum adjuvants such as Alhydrogel are heterogeneous in nature, consisting of 1–10 micrometer-sized aggregates of nanoparticle aluminum oxyhydroxide fibers. To determine whether the particle size and aggregated state of aluminum oxyhydroxide affects its adjuvant activity, we developed a scalable, top-down process to produce stable nanoparticles (nanoalum) from the clinical adjuvant Alhydrogel by including poly(acrylic acid) (PAA) polymer as a stabilizing agent. Surprisingly, the PAA:nanoalum adjuvant elicited a robust TH1 immune response characterized by antigen-specific CD4^+^ T cells expressing IFN-γ and TNF, as well as high IgG2 titers, whereas the parent Alhydrogel and PAA elicited modest TH2 immunity characterized by IgG1 antibodies. ASC, NLRP3 and the IL-18R were all essential for TH1 induction, indicating an essential role of the inflammasome in this adjuvant’s activity. Compared to microparticle Alhydrogel this nanoalum adjuvant provided superior immunogenicity and increased protective efficacy against lethal influenza challenge. Therefore PAA:nanoalum represents a new class of alum adjuvant that preferentially enhances TH1 immunity to vaccine antigens. This adjuvant may be widely beneficial to vaccines for which TH1 immunity is important, including tuberculosis, pertussis, and malaria.

## Adjuvants: Nanoalum gives a boost

Aluminum salt-based adjuvants such as alhydrogel have been a mainstay of vaccines for decades. Christopher B. Fox and colleagues at the Infectious Disease Research Institute in Seattle, USA, investigate the effect adjuvant particle size has on experimental vaccine responses. Shearing conventional micrometer-scale alhyrodogel into nanoparticles is followed by rapid reaggregation; however, the authors show that addition of anionic polymer (PAA) prevents this and results in stable nanoparticles (PAA:nanoalum). Used as an adjuvant with either influenza or TB antigens triggers robust TH1 and IgG2a responses that are superior to alhydrogel even when the latter includes a Toll-like receptor 4 (TLR4) agonist. Interestingly, addition of TLR4 agonist to PAA:nanoalum actually impairs its adjuvanticity. PAA:nanoalum efficacy is nevertheless dependent on the NLRP3 inflammasome suggesting that this novel adjuvant somehow triggers this pathway through some as yet undefined route.

## Introduction

Development of new vaccine adjuvants has entered a golden age of research and development. For decades aluminum salts including aluminum oxyhydroxide and aluminum phosphate (collectively “alum”) were the only adjuvants included in licensed human vaccines in the U.S. The licensure of the squalene oil-in-water emulsion MF59 for influenza vaccines marked the beginning of this new era of adjuvant development. The discovery that Toll-like receptor (TLR) agonists can be utilized to program the adaptive immune response has sparked new developments, particularly adjuvants that robustly augment TH1 immunity. AS04, a combination of the TLR4 agonist MPL and alum, was licensed as a component of vaccines against hepatitis B virus (HBV) and human papillomavirus, making it the first intentional inclusion of a TLR ligand in a licensed vaccine.^1,2^ More recently AS01, a combination of MPL and the saponin QS21, and the TLR9 agonist CpG1018, have achieved licensure as components of herpes zoster and HBV vaccines, respectively. These new adjuvants that promote TH1 immunity are particularly promising for vaccines against malaria, tuberculosis, and pertussis.

These new developments have brought a deeper understanding to the mechanisms of action of adjuvants and the programming of adaptive immunity by the innate immune system. Alum, squalene-in-water emulsions (SE), and QS21 all activate the inflammasome to produce IL-1β and IL-18, important for shaping the adaptive immune response. Inflammasome activation is initiated by the recognition of danger associated molecular pattern (DAMP) containing endogenous molecules by DAMP receptors such as NLRP3. This triggers assembly of the macromolecular inflammasome complex held together by the scaffold protein ASC, leading to the cleavage of pro-caspase 1 and 11 into their active forms. These caspases subsequently cleave pro-IL-1β and pro-IL-18 into their active forms. An emerging paradigm is that combination adjuvants that engage both the TLR signaling cascade and the inflammasome complex are necessary to elicit robust cellular immunity. We and others have reported that TH1 immunity is more effectively activated when a TLR4 ligand is paired with either an SE or QS-21 inflammasome activator, rather than an alum.^3,4^ The reason for this difference is unclear, but may relate to the physical properties of the adjuvant. Alum adjuvants, whether aluminum oxyhydroxide or aluminum phosphate, form aggregated microparticles of heterogeneous sizes, ranging from ~ 0.5–10 µm, referred to as hydrated gels.^5,6^ Conversely, SEs, including MF59 and AS03, are mono-dispersed nanoparticles with an average size of ~ 100 nm. QS21 is similarly formulated in nanoparticle liposomes with a similar ~ 100 nm size. One manufacturing advantage of these nanoparticle adjuvants is that they can be terminally filtrated using a 0.2 µm filter, unlike alum adjuvants which are typically autoclaved.

Recent advances in nanoparticle adjuvant development highlight their potential to be programmed in order to manipulate the immune response. The activity of vaccine adjuvants is partially dictated by particle size by affecting adjuvant uptake and trafficking as well as interactions with the antigen.^6,7^ Alum microparticles remain localized at the site of injection, whereas nano-sized SEs and QS-21 liposomes rapidly traffic to the draining lymph node where they are taken up by the sentinel subcapsular macrophages. The retention of alum at the injection site was initially postulated to boost adjuvant activity, but more recent studies have dispelled that hypothesis.^8,9^ Here, we introduce a top-down manufacturing process—high-pressure microfluidization—to generate aluminum oxyhydroxide nanoparticles, hereupon referred to as nanoalum, using the clinically approved Alhydrogel adjuvant as the precursor. Furthermore, we hypothesized that reducing the particle size of Alhydrogel from micron to nanometer scale would boost the TH1 adjuvant profile when combined with a synthetic TLR4 agonist, SLA (second-generation lipid adjuvant).

## Results

### Production and characterization of stable nanoparticles from Alhydrogel

**Fig. 1:**
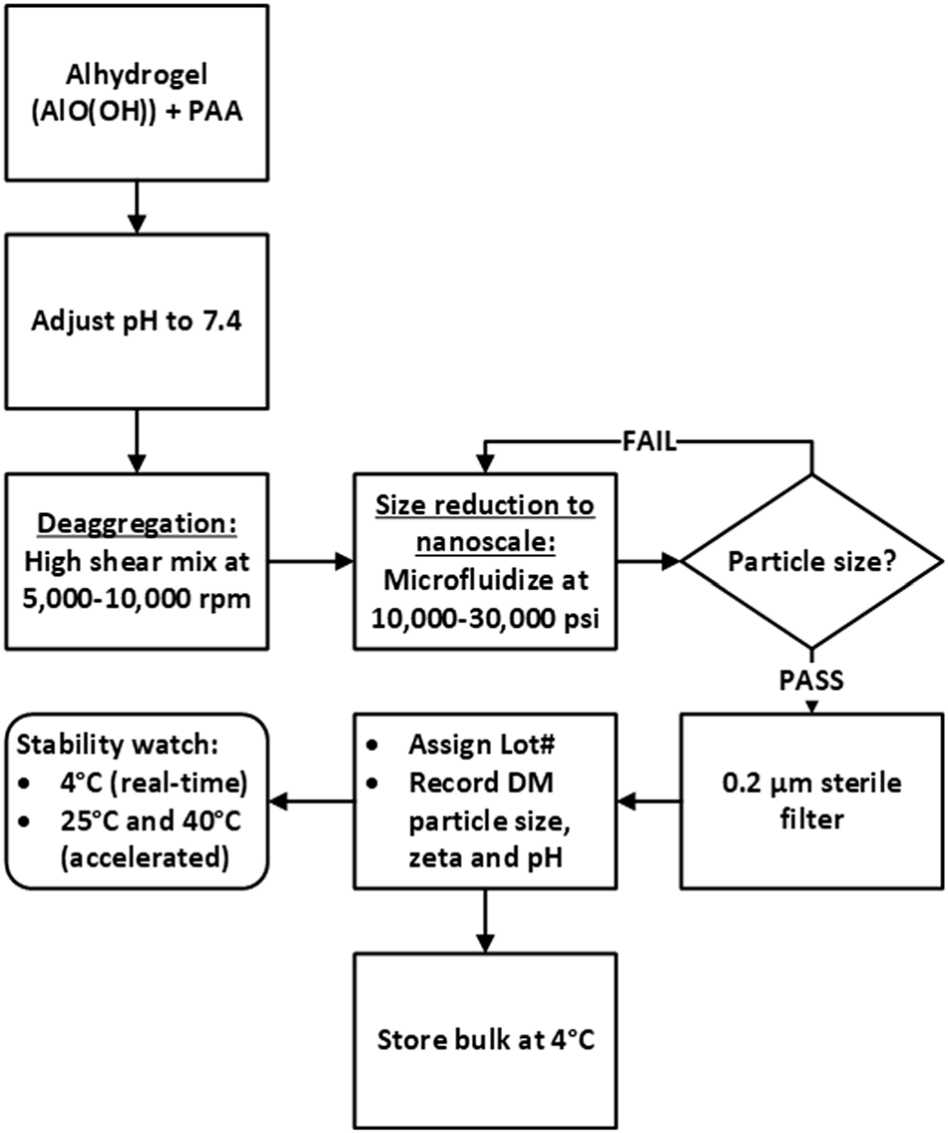
Top down schematic for producing stable nanoalum particles from Alhydrogel

**Fig. 2:**
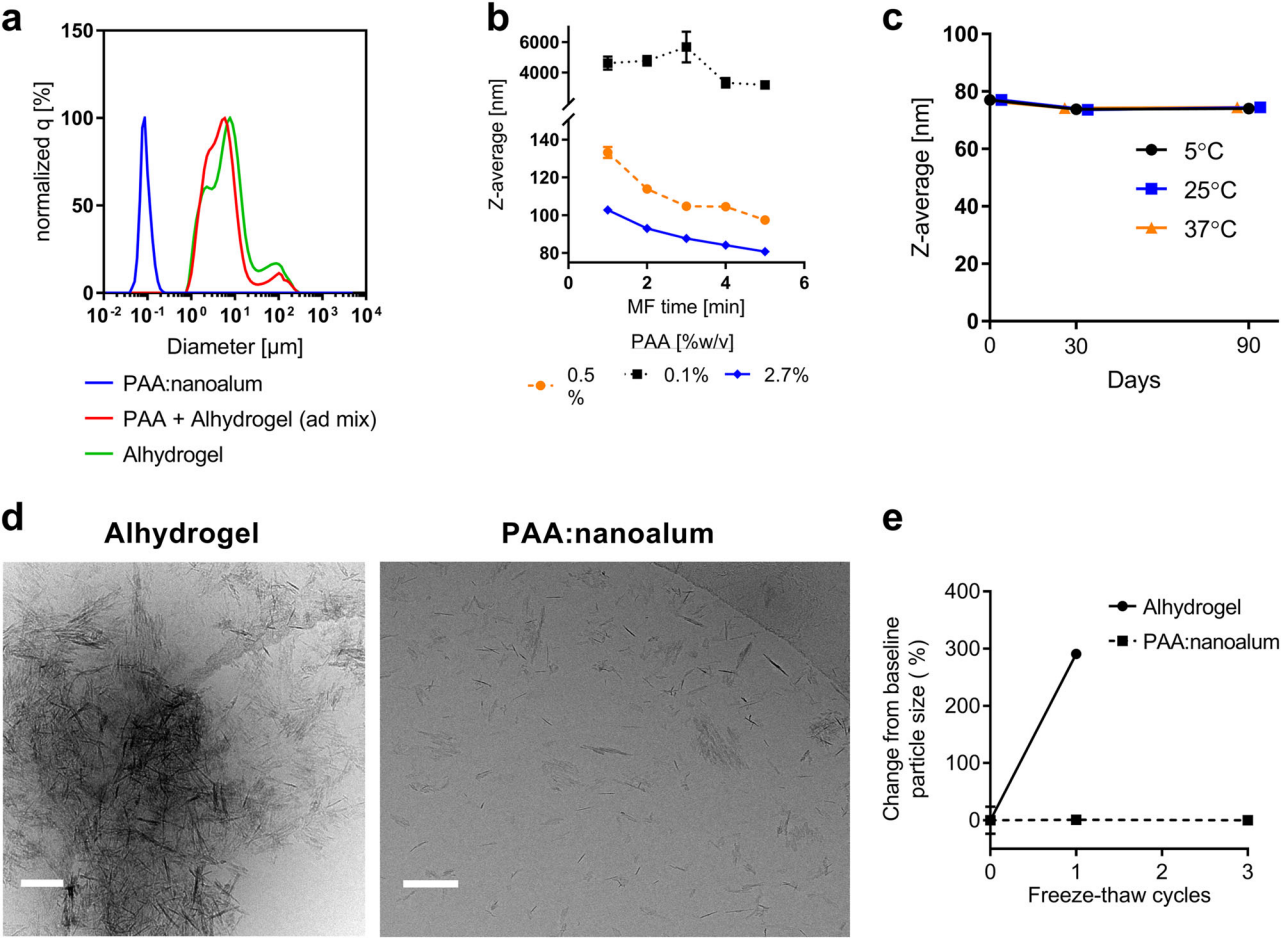
Addition of PAA as a stabilizer produces stable nanoalum particles. **a** The particle size of Alhydrogel, Alhydrogel mixed with PAA prior to sonication and microfluidization, and PAA:nanoalum were determined by dynamic light scattering (DLS) or laser diffraction. **b** Increasing the amount of PAA at the start of the process or increasing the number of passes through the microfluidizer results in smaller PAA:nanoalum particles. **c** PAA:nanoalum retains the original particle size for at least 3 months at 5, 25, or 37 °C as determined by DLS. **d** Cryo-TEM images of Alhydrogel on the left and PAA:nanoalum on the right reveal a highly aggregated and mono-dispersed state, respectively. Scale bars, 100 nm. **c** Cryo-TEM images of Alhydrogel on the left and PAA:nanoalum on the right reveal a highly aggregated and mono-dispersed state, respectively. **d** PAA:nanoalum retains the original particle size for at least 3 months at 5, 25, or 37 °C as determined by DLS. **e** Unlike Alhydrogel, PAA:nanoalum particle size is stable for at least three free-thaw cycles. Data are representative of three experiments with three replicates each. The means and standard deviations are shown

The clinical alum adjuvant Alhydrogel consists of fibrous nanoparticles with average dimensions of 4.5 × 2.2 × 10 nm, which form aggregates with a broad size distribution from 0.5–10 microns in suspension.^10^ These particles can be temporarily dispersed using sonication or other high shear methods, but quickly reaggregate over a matter of hours (Supplementary Fig. 1). To prevent reaggregation we introduced a low molecular weight anionic polymer, 2 kDa poly(acrylic acid) (PAA), prior to the microfluidization step (Fig. 1). PAA has been included in a number of food and drug products and a 450 kDa cross-linked PAA is the basis of the veterinary adjuvant Carbopol.^11–13^ Inclusion of the PAA before microfluidization produced stable nanoparticles with Z-average hydrodynamic diameter (*d*_H_) of 68 nm with a monodisperse size distribution (polydispersity index = 0.14) (Fig. 2a). Unlike Alhydrogel, PAA:nanoalum was amenable to sterile filtration using a 0.2 µm filter. Increasing the ratio of PAA to aluminum and/or the number of passes through the microfluidizers resulted in smaller PAA:nanoalum particles, between 75 and 110 nm (Fig. 2a, b). Cryo-TEM imaging of PAA:nanoalum revealed uniformly distributed rod-shaped aluminum oxyhydroxide particles, whereas Alhydrogel displayed large and heterogeneous particle aggregates (Fig. 2c). Unlike microfluidized Alhydrogel, the nanoparticle size of PAA:nanoalum was stable, as indicated by a lack of particle size growth over 3 months when stored at 25 or 37 °C (Fig. 2d) and over 1 year at 5 °C. Moreover, in contrast to Alhydrogel, the PAA:nanoalum formulation remained stable even after multiple freeze-thaw cycles (Fig. 2e).

### PAA:nanoalum promotes TH1 immunity without a TLR4 agonist

**Fig. 3:**
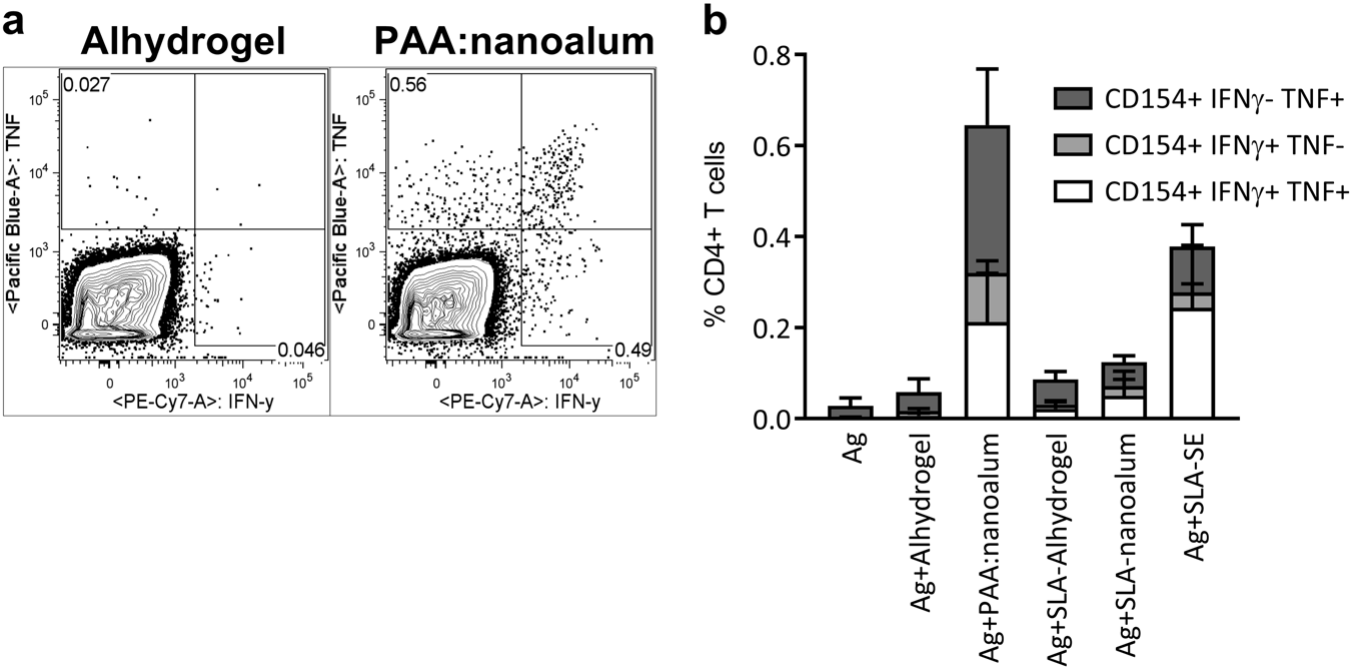
PAA:nanoalum promotes TH1 immunity without exogenous TLR4 agonistsl. C57Bl/6 mice (five per group) were immunized twice with 0.5 µg of ID93 alone or adjuvanted as indicated. One week after a homologous booster immunization the frequency of antigen-specific CD4 T cells in the spleen was determined by intracellular cytokine staining after stimulation with the immunizing antigen. **a** A representative FACS plot is shown for antigen stimulated cells from the Alhydrogel and PAA:nanoalum animal (left and right, respectively). **b** Distribution of CD154, IFN-ƴ and TNF producing CD4 T cells. Representative FACS gating is shown in Supplementary Fig. 3A. *N* = 5 animals per group. Data are representative of three experiments with similar results. Bars are drawn to the means with whiskers to the standard deviations

To test our hypothesis that reducing the particle size of alum would augment its synergy with a TLR4 agonist to promote TH1 immunity, we immunized C57BL/6 mice twice (prime and boost 3 weeks apart) with the recombinant TB vaccine antigen ID93 alone or adjuvanted with Alhydrogel or PAA:nanoalum in presence or absence of the synthetic TLR4 agonist SLA. As a positive control, we used SLA-SE, a combination adjuvant of SLA formulated in a squalene-in-water nanoemulsion (SE) with a mean particle size of 100 nm. All of the immunizations were well tolerated at both the prime and boost immunization, as we did not note any local injection site reactogenicity or altered animal behavior after the immunizations. TH1 responses were determined by CD4^+^ T cells expressing CD154 (CD40L) as well as either or both of the TH1-associated cytokines IFN-γ and/or TNF upon antigen exposure. SLA-Alhydrogel modestly augmented the ID93-specific CD4^+^ T cell response, as measured with the frequency of antigen-specific T cells positive for TH1 cytokines, 1 week after the booster immunization (Fig. 3). SLA-SE significantly enhanced the TH1 response compared to Alhydrogel (*P* < 0.01). Replacing Alhydrogel with PAA:nanoalum in combination with SLA did not improve the TH1 responsiveness, indicating that the difference in TH1 programming potential between SLA-SE and SLA-alum is not simply due to a difference in adjuvant particle size. Surprisingly, the PAA:nanoalum adjuvant without the TLR4 agonist component promoted a robust TH1 immune response, that was greater (*P* < 0.01) than the response elicited by SLA-SE (Fig. 3). Thus PAA:nanoalum on its own was sufficient to drive a potent TH1 response.

**Fig. 4:**
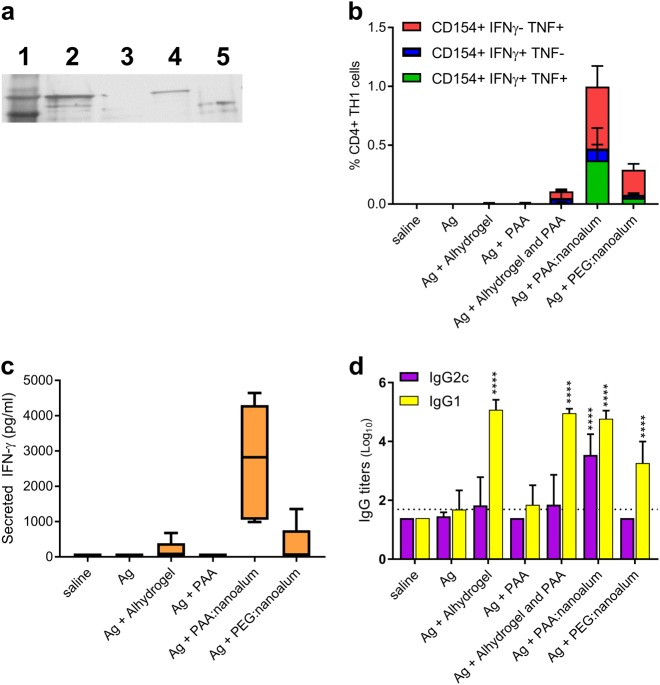
Augmentation of TH1 immunity is not a general property of nanoalum formulations or free PAA. **a** ID93 was mixed with Alhydrogel, PAA, or PAA:nanoalum for detected by silver stain before and after centrifugation to pellet the alum particles. Lane 1: ladder, 2: ID93, 3: ID93 + Alhydrogel, 4: ID93 + PAA:nanoalum 5: ID93 + PEG:nanoalum. The full gel is shown in Supplementary Fig. 2. **b**–**d** C57Bl/6 mice (five per group) were immunized twice with 0.5 µg of ID93 alone or adjuvanted with Alhydrogel, PAA, PAA:nanoalum, or PEG:nanoalum. **b** One week after the second immunization, splenocytes were isolated and either unstimulated or stimulated with the ID93 protein in the presence of brefeldin A for 8 h at 37 °C. Cells were then stained for surface expression of CD4 and CD44, as well as intracellular expression of IFN-ƴ, TNF and/or CD154. **c** Splenocytes from immunized mice were stimulated with the ID93 protein and assessed for secretion of IFN-ƴ. **d** Serum was collected from immunized animals 3 weeks after the first immunization and assessed for ID93 binding antibody titers by ELISA for IgG1 and IgG2 subclasses. ***(*P* < 0.001), ****P < 0.0001 relative to the antigen alone group as determined by one-way ANOVA with Dunnett’s correction for multiple comparisons. Representative FACS gating is shown in Supplementary Fig. 3A. Data are representative of three experiments with similar results. **a** and **d** Bars are drawn to the means with whiskers to the standard deviations. **c** The line is drawn at the mean, the box at the interquartile range and the whiskers at the min and max

PAA:nanoalum has a negative zeta potential (ZP) of −18.0 ± 2.8 mV at pH = 7.4, unlike Alhydrogel, which has a positive surface charge (ZP = + 9.6 ± 1.0 mV at neutral pH). Thus, we assessed the potential for binding to a recombinant vaccine TB antigen, ID93, which has a net negative charge at neutral pH (pI = 5.03). Adsorption was determined by the loss of detectable ID93 in the supernatant after centrifugation to pellet the Alyhdrogel or PAA:nanoalum. Alhydrogel bound ID93 as demonstrated by a loss of detectable ID93 after centrifugation of the ID93 and Alhydrogel mixture (Fig. 4a, lane 3). Conversely free ID93 was detectable after ultracentrifugation of the ID93 and PAA:nanoalum mixture (lane 4) indicating a lack of ID93 binding. To determine whether this loss of antigen binding capacity of the PAA:nanoalum was due to the change in zeta potential or increased surface availability, we produced a second nanoalum using the process outlined in Fig. 1, replacing the PAA with PEG(5000)-DSPE as the stabilizing polymer. The resulting PEG:nanoalum had a similar particle size (70 nm) and polydispersity index (0.15) and was stable at 5 °C for over 1 month. This PEG:nanoalum had a neutral net surface charge of −0.7 ± 0.3 mV and bound ID93 (Fig. 4a, lane 5), suggesting that surface charge, not particle size, was the key determinant in antigen binding. Thus, coating the surface of the aluminum particles by the PAA polymers, which resulted in a negative zeta potential, prevented the electrostatic binding of ID93.

Previous studies of nanoparticle alum adjuvants have not reported this striking TH1 promoting potential, therefore we sought to determine whether this is an unreported generic property of nanoparticle alum adjuvants or specific to the PAA:nanoalum. Unlike the PAA:nanoalum, PEG:nanoalum did not induce any appreciable TH1 response after two immunizations, indicating that TH1 promotion is not a generic property of nanoparticle alum adjuvant, but rather unique to the PAA:nanoalum (Fig. 4b). Large molecular weight ( > 300 kDa) cross-linked PAA polymers have TH1 promoting adjuvant properties on their own and are the basis of the veterinary adjuvant Carbopol, which contains 450 kDa cross-linked PAA.^11–13^ On its own, an equal molar amount of 2 kDa free PAA used to produce the PAA:nanoalum was not sufficient to promote TH1 immunity, suggesting that this adjuvant activity is due to the PAA:nanoalum, not just the presence of PAA. Importantly an admixture of PAA and Alhydrogel was not sufficient to recapitulate the adjuvanticity of PAA:nanoalum indicating that the nanoparticle form was also crucial for this activity. Similar to the intracellular cytokine profile, splenocytes from the PAA:nanoalum adjuvanted animals, but not the other groups, secreted a substantial amount of IFN-ƴ upon stimulation with the immunizing antigen (Fig. 4c). In addition to eliciting a robust TH1 cellular immune profile, PAA:nanoalum drove a balanced, high-titer, IgG1 and IgG2c serum response to ID93 (Fig. 4d). In contrast, Alhydrogel with or without admixed PAA strongly skewed the humoral response to the IgG1 subclass, that was not different from the response to PEG:nanoalum. The free PAA adjuvant did not significantly boost antibody titers. Taken together these results indicate that the TH1 and balanced antibody production with PAA:nanoalum depends on both the PAA and the nanoalum.

### PAA:nanoalum creates a TH1 competent innate immune response and activates the inflammasome

**Fig. 5:**
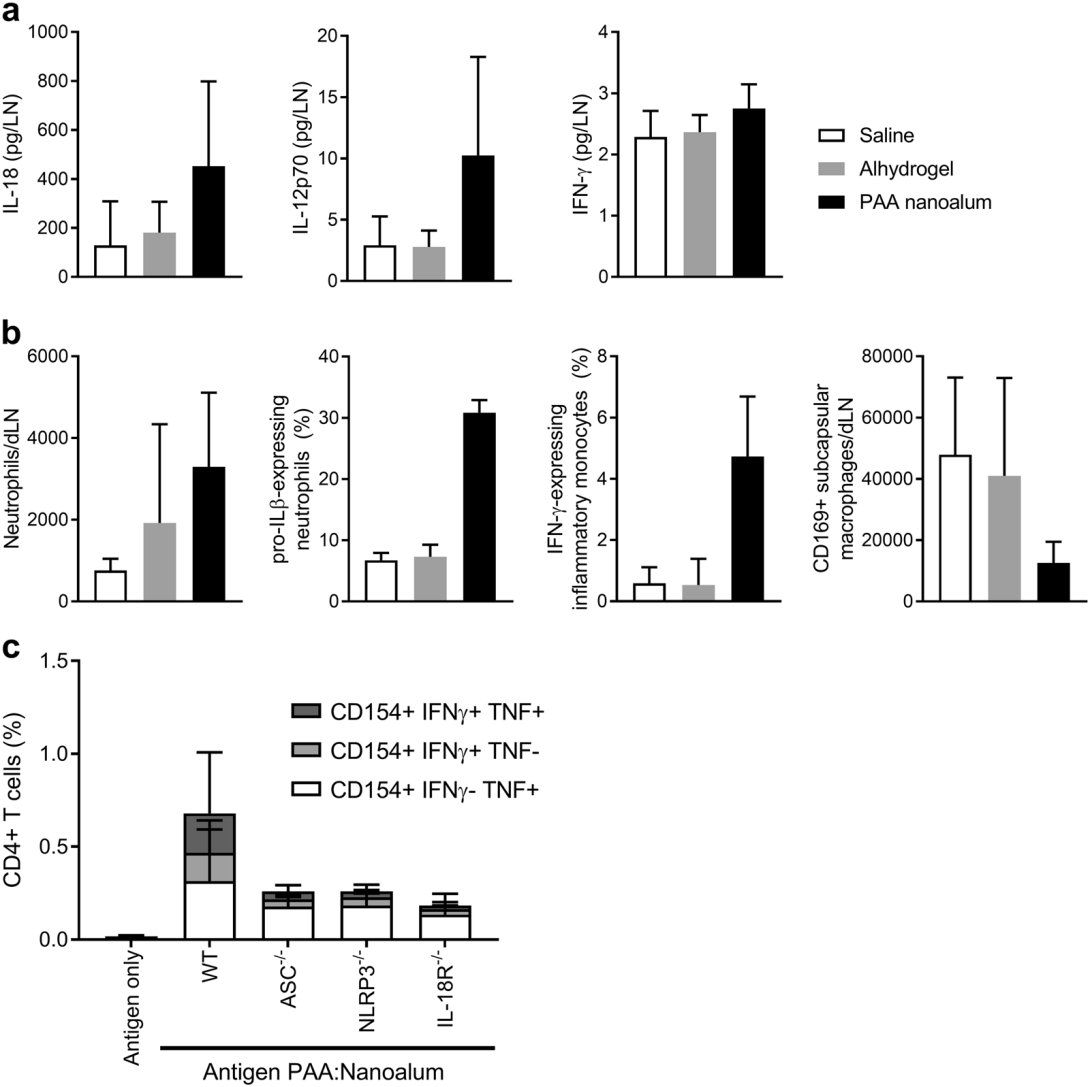
PAA:Nanoalum creates an immunocompetent lymph node environment and activates the inflammasome. **a** The amount of IL-18, IL-12p70 and IFN-γ in the draining lymph node 6 h after immunization with saline, Alhydrogel or PAA:nanoalum. **b** At the same time, the numbers of neutrophils and subcapsular macrophages were enumerated and intracellular IL-1β and IFN-γ expression were determined by ICS. **c** Antigen-specific CD4 T cells were determined in wild-type (WT), ASC-/-, NLRP3-/-, or IL-18R-/- mice 1 week after the boosting immunization with either antigen alone or antigen adjuvanted with PAA:nanoalum using ICS. Representative FACS gatings are shown in Supplementary Fig. 3A, B. *N* = 5 mice per group. The data are representative of two experiments with similar results. Bars are drawn to the means with whiskers to the standard deviations

We previously found that the TH1 and antibody augmenting ability of GLA-SE depended on activation of the inflammasome to produce IL-1β, IL-18 as well as IL-12 and innate IFN-γ.^14-16^ Additionally, subcapsular macrophages were essential to capture the adjuvant in the draining lymph node (dLN) rapidly after immunization. These cells are lost from the dLN after adjuvanted immunization or infection.^17^ We assessed the innate immune response in the dLN 6 h after immunization to determine if a similar activation of the inflammasome was induced by PAA:nanoalum. Compared to Alhydrogel, PAA:nanoalum produced a substantial increase in IL-18 and IL-12p70 with a trend towards increases in IFN-γ in the draining LN, although the latter did not reach statistical significance (Fig. 5a). There was also an augmented influx of neutrophils, a greater fraction of which contained pro-IL-1β, an increased frequency of inflammatory macrophages producing IFN-γ, and a substantial loss of subcapsular macrophages (Fig. 5b). All of these changes caused by PAA:nanoalum are similar to our previous findings with GLA-SE, suggesting a similar mode of action is shared between these two TH1-augmenting adjuvants.

Particulate adjuvants including squalene emulsions (e.g., SE, MF59, or AS03) activate the inflammasome to produce bioactive IL-1β and IL-18. The role of the inflammasome activation in the action of alum adjuvants remains controversial.^18–21^ We have also found that IL-1R, IL-18R and the inflammasome assembly protein ASC are essential for the TH1 activity of GLA-SE.^14,16^ In order to assess whether the inflammasome played a crucial role in the adjuvanticity of nanoalum, we immunized WT, ASC^-/-^, IL-18R^-/-^, and NLRP3^-/-^ mice with antigen + PAA:nanoalum. Abrogation of the NLRP3—ASC—IL-18 axis substantially decreased both the TH1 CD4^+^ T-cell and B-cell response indicating that this inflammasome cascade was essential for PAA:nanoalum activity (Fig. 5c).

### Nanoalum is a superior adjuvant to microparticle Alhydrogel for producing protective immunity against influenza

**Fig. 6:**
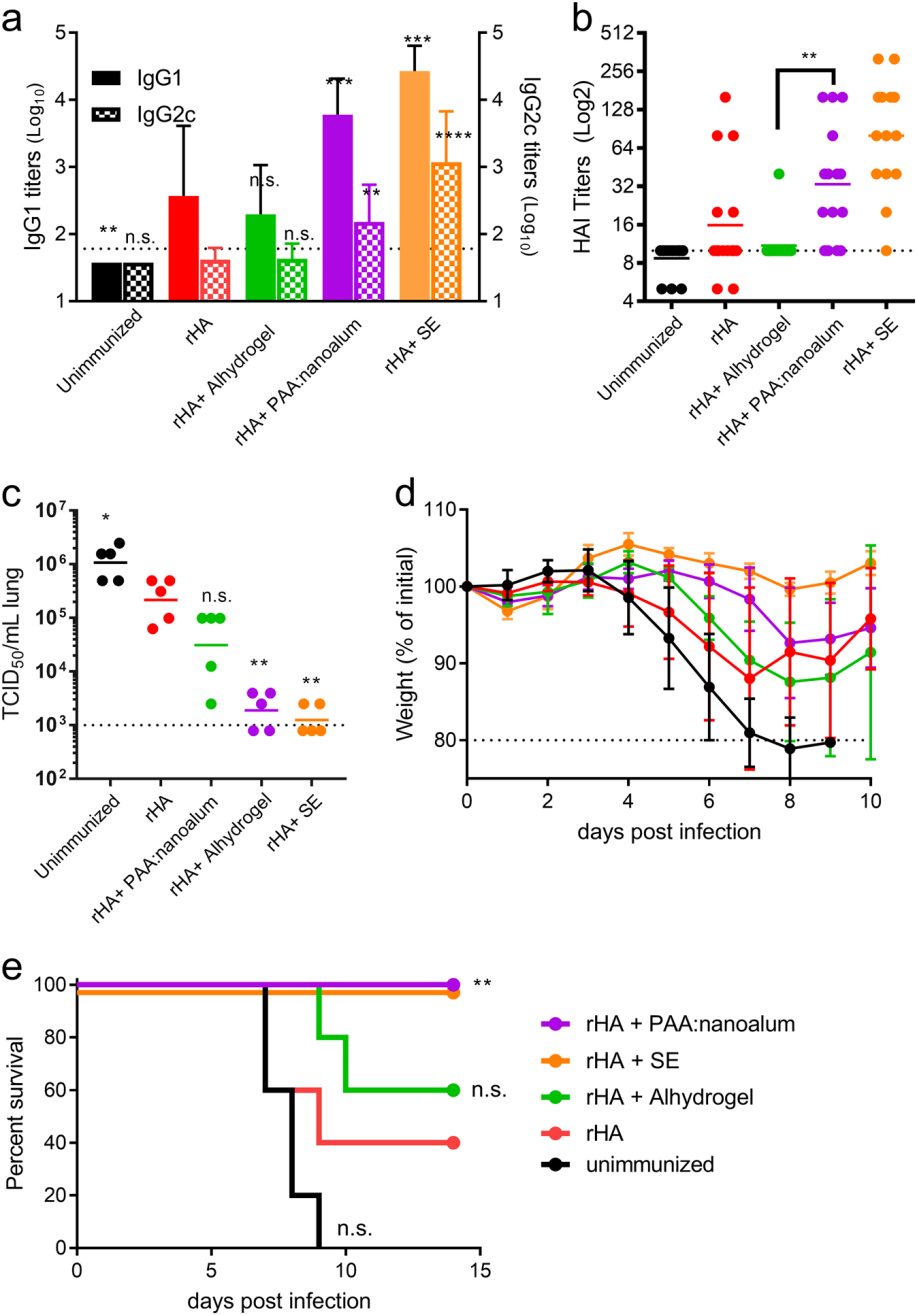
Nanoalum significantly augments the immunogenicity and protective efficacy of a flu vaccine. C57Bl/6 mice were immunized with recombinant H1 antigen alone or adjuvanted as indicated. Three weeks after the immunization serum antibody titers **a** and HAI titers **b**. One week later the animals (*N* = 10/group) were intranasally challenged with 25LD50 of the PR8 strain of H1N1 influenza. Viral titers in the lungs were determined at day 4 after challenge (*N* = 5/group) **c**. The remaining 5/group were monitored for weight loss **d** and mortality **e**. **a**, **b**, and **c** *, **, ***, and **** indicate *P* < 0.05, 0.01, 0.001, and 0.0001 as determined by one-way ANOVA with Dunnett’s correction for multiple comparisons, compared to the rHA alone control, respectively, unless the comparison is otherwise indicated. **e** **indicates *P* < 0.01 as determined by Log-rank (Mantel-Cox) test compared to rHA alone. Data are representative of two individual experiments. The **a**, **c**, and **d** means and standard deviations or **b** geometric means are shown

Microparticle aluminum adjuvants including Alhydrogel are generally regarded as ineffective in augmenting split inactivated or subunit influenza vaccines.^22^ In this gap, a number of adjuvants have been developed to increase the immunogenicity and safety of flu vaccines, primarily oil-in-water emulsions such as MF59 and AS03 that are included in licensed flu vaccines.^23,24^ In head-to-head clinical trials, oil-in-water emulsions consistently out-perform alum adjuvants for a number of flu vaccines including recent trials of vaccines against H5 and H7 avian influenzas. Here, we sought to determine if PAA:nanoalum would be a more effective alum adjuvant than microparticle Alhydrogel for flu vaccines. Three weeks after a single immunization with a recombinant H1 protein antigen (rHA) addition of the nanoalum adjuvant significantly augmented serum IgG1 and IgG2 responses, whereas Alhydrogel had no benefit (Fig. 6a). Serum hemagglutination (HAI) titers are the accepted correlate of protection for influenza vaccines. Importantly, the nanoalum adjuvanted animals had a significantly higher HAI titer compared to the Alhydrogel adjuvanted group (Fig. 6b). Both serum antibody and HAI titers elicited with nanoalum were close to those achieved with a stable oil-in-water nanoemulsion (SE), the gold standard class of flu vaccine adjuvant. Correlating with the high HAI titers, four days after intranasal challenge with the PR8 strain of H1N1 influenza, lung viral titers were significantly lower in animals receiving the nanoalum adjuvanted vaccine, whereas the Alhdyrogel adjuvant had no significant benefit (Fig. 6c). The viral titer reduction achieved with the nanoalum adjuvanted vaccine was similar to that achieved with the SE adjuvant. Adjuvanting with either nanoalum or SE ameliorated morbidity in the infected mice, with 100% survival in both vaccinated groups (Fig. 6d, e). By comparison protein alone protected 40% of animals and Alhydrogel only boosted this to 60% (Fig. 6e). Taken as a whole, the nanoalum adjuvant was substantially more effective than Alhydrogel in boosting the immunogenicity and protective efficacy of the recombinant protein antigen.

## Discussion

Next-generation vaccine development for many infectious diseases including influenza, HIV, tuberculosis and malaria, is refocusing from attenuated or inactivated first generation vaccine technologies to newer and safer defined-subunit vaccine approaches. However, many of these subunit vaccines fail to induce effective immunity without the addition of adjuvants. First developed in the 1920’s, aluminum salts (alum) are the most commonly used human vaccine adjuvants, and are included in vaccines for polio, hepatitis A and B, anthrax, pneumococcus, diphtheria, tetanus, pertussis, and others.^5,25^ Alum adjuvants have an extensive record of safe use, and they effectively augment TH2 cellular immunity and antibody titers. Despite their long history of use as vaccine adjuvants, aluminum salts are structurally complex and heterogeneous, making them a challenge to characterize for quality control, especially after mixing with vaccine antigens. This complexity is compounded by the fact that there are multiple types of aluminum salts available with distinct properties (e.g., size, surface charge, crystallinity), including aluminum phosphate, aluminum hydroxyphosphate sulfate, and aluminum oxyhydroxide.^5^ Moreover, cold chain excursions resulting in freezing of vaccines containing aluminum salts disrupt the gel structure and reduce adjuvant activity, making them unsuitable for use. From an immunologic perspective, alum-adjuvanted vaccines fail to induce effective TH1 or TH17 cellular immunity, which are important for vaccines against influenza, pertussis, malaria, tuberculosis, and other diseases.^26–28^ Alum-adjuvanted acellular pertussis (aP) vaccines also fail to generate long-lived protective antibodies.^29,30^ Indeed, seasonal influenza vaccines are preferentially adjuvanted with oil-in-water emulsions due to the increased efficacy associated with the higher titer of functional antibodies.

Several studies have shown potential benefits of nanoscale alum over conventional alum. *B. anthracis* adsorbed on aluminum hydroxide nanoparticles induced stronger and more durable antigen-specific IgG levels in mice compared to micron-sized aluminum hydroxide particles.^31^ Similarly rod-shaped aluminum oxyhydroxide nanoparticles more effectively augmented serum IgG titers in vivo compared to micron-sized aluminum oxyhydroxide crystals.^32^ Previous studies investigating the effect of particle size on alum adjuvanticity employed molecular aluminum precursors to manufacture nanoparticles; commonly referred to as the LaMer method, nanoparticles first form in a nucleation step and undergo growth until the precursor material is used.^33^ However, in most cases no comparison was made to clinical aluminum salt adjuvants such as Alhydrogel, making it difficult to interpret the value of the formulations.^31,32,34,35^ While the LaMer and other bottom-up synthesis methods provide excellent control over nanoparticle size and crystal structure, large-scale manufacturing to support clinical translation is challenging.

Here, we have demonstrated that physicochemical modifications of alum, including reducing the stable particle size to the nanometer scale, can greatly influence qualitative immune response patterns. Specifically, rendering the clinical alum adjuvant Alhydrogel as nanoparticles with the addition of a low molecular weight PAA polymer to prevent reaggregation yielded an alum adjuvant that promoted TH1 immune responses. The magnitude of this response was higher than that achieved with the synthetic TLR4 agonist SLA formulated in a 2% squalene nanoemulsion. In conjunction with the multifunctional TH1 cells producing CD154, IFN-γ and /or TNF PAA:nanoalum elicited a robust and balanced generation of IgG1 and IgG2 antibodies. That a similarly sized PEG:nanoalum produced via the same method did not augment TH1 or IgG2 immunity points to a unique contribution of the PAA polymer. While it has been reported that larger complexes of PAA such as Carbopol can be Th1 biasing, the type used in this report does not on its own bias to Th1. One could speculate that the Th1 bias is achieved via presentation on the surface of the nanoalum thereby mimicking larger Carbopol-like molecules.

A thorough understanding of the mechanisms of action of any new adjuvant is crucial for further rational improvements and informed clinical evaluation. Many adjuvants act as ligands for innate recognition receptors (PRRs) that recognize pathogen or danger associated molecular patterns (PAMPs or DAMPs) and activate innate responses.^36^ PRR signaling induces production of specific cytokines and chemokines that determine which immune cells are recruited to the site of injection, and ultimately will shape the adaptive responses. Unlike PAMPs, which are unique to classes of pathogens (e.g., LPS on gram-negative bacteria that triggers TLR4 or dsRNA of certain virus families that triggers TLR3), endogenous molecules containing DAMPs, such as extracellular ATP, uric acid, dsDNA, and HSP70, released from damaged or dying cells act as danger signals when they accumulate in non-steady state locations, activating the inflammasome.^37–39^ For instance, the squalene oil-in-water emulsion, MF59, enhances immunity by driving release of extracellular ATP, which activates the inflammasome cascade to produce the inflammatory cytokines IL-18 and IL-1β.^36,40,41^ This leads to attraction of neutrophils and monocytes to the site of injection and increases antigen uptake.^42^ Alum acts through a variety of danger signals, such as uric acid,^18^ heat shock protein 70,^43^ and DNA released from necrotic cells exposed to alum.^44^ These events trigger innate cell recruitment (macrophages, monocytes, and granulocytes) to the injection site, and enhances antigen uptake by dendritic cells.^45^ Activation of the NLRP3 inflammasome to produce bioactive IL-1β has also been implicated in the adjuvanticity of alum.^46^ GLA-SE, another TLR4 agonist, lipid adjuvant formulated in squalene oil-in-water emulsion, signals through the NLRP3 inflammasome driven IL-18 and IL-1β to promote TH1 and humoral immunity.^14,15^

Little is known about the mechanisms by which nanoparticle alum adjuvants augment immunity, particularly T-cell responses. The more potent adjuvant effect of alum nanoparticles has been attributed to antigen uptake by DCs and macrophages, which was significantly increased when *B. anthracis* was coupled to nanoscale aluminum particles rather than micrometer-scale particles.^31^ The adjuvant activity was dependent on NLRP3 inflammasome activation, increased release of uric acid, and increased IL-1β secretion.^35,47^ Compared to micron-sized aluminum oxyhydroxide, rod-shaped aluminum nanoparticles induced significantly enhanced reactive oxygen species production and a higher IL-1β secretion, dependent on the activation of the NLRP3 inflammasome. These alum nanorods also induced IL-6 and IL-12 production in vitro and antigen uptake in vivo.^32,34^ The importance of activation of the inflammasome via NLRP3 to alum adjuvanticity is a debated topic and may depend on the type of alum used. We found that the Alhydrogel based PAA:nanoalum required NLRP3 inflammasome activation to produce IL-18 and promote TH1 immunity. Compared to microparticle aluminum oxyhydroxide, PAA:nanalum increased the innate expression of IL-12p70, IFN-γ, pro-IL-1β and disappearance of subcapsular macrophages from the draining lymph node, suggesting a different mechanism of action between nanoalum and microparticle Alhydrogel.

In summary, we have developed an exciting new class of alum adjuvant that potently programs TH1 and IgG2 immunity by activating the inflammasome pathway and modifying the innate immune environment. We achieved this by producing stable nanoparticles by dispersing the clinical alum adjuvant Alhydrogel in the presence of a small polymer, PAA. This adjuvant may have a number of clinical applications to improve on suboptimal vaccines that employ an alum adjuvant, such as acellular pertussis vaccines, vaccines for which traditional microparticle alum adjuvants have proven ineffective, such as influenza, and for diseases for which no vaccine exists, such as tuberculosis.

## Methods

### Nanoalum synthesis

To prepare a 200 ml batch of PAA:nanoalum, 10.8 g of 50% w/v 2000 Da poly(acrylic acid) solution (Sigma-Aldrich) was added to 180 ml of 10 mg Al/ml Alhydrogel® “85” (Sergeant Adjuvants) gel and mixed in a 500 ml sterile PETG bottle (Nalgene). The mixture was adjusted to pH 7 with 1–10 M NaOH solution and then QS to 200 ml with DI water. A silverson high shear mixer was used to homogenize the mixture at 5000–10,000 rpm for 5–10 min to break up large AlO(OH) aggregates. To further reduce particle size down to the nanometer scale, the Alhydrogel-PAA mixture was microfluidized in the M110P microfluidizer (Microfluidics, Inc.) at 30,000 psi for ten passes. The re-circulating water bath, used to control the M110P’s cooling coil temperature, was set to 4 °C. The target particle size of the microfluidized sample (60–80 nm) was confirmed with dynamic light scattering (DLS; Zetasizer ZS, Malvern Instruments) before sterile filtering with 0.2 µm PES membrane. Particle size, zeta potential and pH of the filtered PAA:nanoalum material were recorded at the date of manufacture. Unless otherwise specified, all studies were performed with a PAA:nanoalum preparation containing 27 mg/ml 2 kDa PAA and 9 mg/ml elemental aluminum.

### Particle size and Zeta potential measurements

Hydrodynamic diameter of nanoparticle formulations was determined using the Dynamic Light Scattering (DLS) technique (Zetasizer Nano ZS, Malvern Instruments, Ltd.). Formulations were diluted 1:10 with water in triplicate and measured in a disposable polystyrene cuvette (SOP parameters: material RI = 1.59, dispersant RI (water) = 1.33, *T* = 25 °C, viscosity (water) = 0.887 cP, measurement angle = 173° backscatter, measurement position = 4.65 mm, automatic attenuation). Particle size distribution of Alhydrogel adjuvant was measured using laser diffraction (LA-960 Laser Particle Size analyzer, Horiba Scientific). Measurement parameters were as follows: material RI = 1.59, dispersant RI = 1.33, *T* = 25 °C, red laser *λ* = 655 nm (90–95% transmittance), blue laser *λ* = 405 nm (80–90% transmittance), circulation speed = 5, continuous agitation speed = 3, ultrasound off during measurement. For zeta potential measurement, formulations were diluted 1:50 in triplicate and loaded in a disposable DTS1070 (Malvern instruments, Ltd.) folded capillary cell. The following SOP parameters were used: material RI = 1.59, dispersant RI (water) = 1.33, viscosity (water) = 0.887 cP, *T* = 25 °C, automatic attenuation and voltage selection. The intensity-weighted Z-average diameter, PDI and zeta potential values for each formulation, averaged from three measurements/replicate (nine total measurements), were recorded.

### Freeze-thaw stability

PAA:nanoalum and Alhydrogel formulations were frozen in a dry ice/acetone bath and subsequently thawed in a 37 °C water bath. The freeze-thaw cycle was repeated three times; after each cycle, formulations were visually inspected for sedimentation. Particle size of PAA:nanoalum was measured with DLS (Malvern Instruments, Zetasizer ZS) while Alhydrogel was measured with laser diffraction (Horiba Scientific, LA-960).

### Cryo-electron microscopy imaging

To evaluate the impact of particle size reduction via microfluidization on the morphology of nanoalum formulations, we sent samples for cryo-Transmission Electron Microscopy (cryo-TEM) imaging analysis on a vendor service basis to NanoImaging Services.

### Mice, immunizations, and tissue harvesting

Female wild-type (WT) C57BL/6, NLRP3^-/-^, and IL-18R^-/-^ mice aged 6–10 weeks were purchased from the Jackson Laboratory. ASC^-/-^ were a kind gift of Amy Hise (Case Western Reserve University, USA) and were bred in-house. All strains were on the C57BL/6 background and maintained in Specific Pathogen Free conditions. All animal experiments and protocols used in this study were approved by the Infectious Disease Research Institute’s Institutional Animal Care and Use Committee. Mice were immunized twice, 3 weeks apart via an intramuscular injection in the calf muscles of hind limb with 0.5 µg of ID93 or 0.1 µg rHA recombinant protein.^48^ Adjuvants were added as follows per dose: SE (2%), SLA (5 µg), Alhydrogel (100 µg aluminum) nanoalum (100 µg aluminum), and/or PAA (2.7 µg). For innate immune responses, draining inguinal lymph nodes were collected 6 h after immunization. For adaptive immune responses, spleens were collected in RPMI 7 days after the second immunization. Cells suspensions were obtained by manual disruption. Red blood cells contained in spleens were lysed using the Red Blood Cell Lysis Buffer (ThermoFisher). Total viable cells were counted (Guava Technologies, Millipore) and plated at 2 × 10^6^ cells/well in round-bottom 96-well plates.

### Flow cytometry

For intracellular cytokine staining, cells were stimulated for 2 h with media (RPMI 1640 + 10% FCS) or ID93 (10 µg/ml) at 37 °C and subsequently incubated with Brefeldin A for an additional 8 h at 37 °C. Cells were surface stained with CD4 BV650 (clone RM4–5; BioLegend Cat# 100551), CD8 BV786 (clone 53–6.7; BioLegend Cat# 100742), B220 BV510 (clone RA3–6B2; BioLegend Cat# 103248), CD11b BV510 (clone M1/70; BD Biosciences Cat# 562950), and CD44 APC-eF780 (clone IM7; eBioscience Cat#47-0441-82), together with Fc receptor block (anti-CD16/32 antibody; BD Biosciences Cat# 553142) (all 1:200), followed by permeabilization with Cytofix/Cytoperm (BD Biosciences) and intracellular staining with CD154 PerCP-eF710 (clone MR1; eBioscience Cat# 46-1541-82), TNF eF450 (clone MP6-XT22; eBioscience Cat# 48-7321-82), and IFN-γ PE-Cy7 (clone XMG1.2; eBioscience Cat# 25-7311-82) all at 1:200. Cells were gated as singlets > lymphocytes > CD4 + CD8- B220- CD11b- > CD44 + > cytokine + (Supplementary Fig. 3A).

For innate staining, draining lymph node cells were stained with Ly6G FITC (clone 1AB; BioLegend Cat# 127606), Ly6C PerCP-Cy5.5 (clone HK1.4; BioLegend Cat# 128012), MHC-II eF450 (clone M5/114.15.2; eBioscience Cat #48-5321-82), CD169 (Siglec-1) BV605 (clone 3D6.112; BioLegend Cat# 142413), CD8 BV510 (clone 53-6.7; BioLegend Cat# 103248), CD4 BV785 (clone RM4-5; BioLegend Cat# 100551), B220 BV785 (clone RA3–6B2; BioLegend Cat# 103245), CD11b-AF700 (clone M1/70; eBioscience Cat# 56-0112-82), CD11c APC-Cy7 (clone N418; eBioscience Cat# 47-0114-82) and NK1.1 PE (clone PKI136; BioLegend Cat# 108708) (all at 1:100). After permeabilization, cells were stained with pro-IL-1β APC (clone NJTEN3; eBioscience Cat# 17-7114-80) and IFN-γ PE-Dazzle 594 (clone XMG1.2; BioLegend Cat# 505846) (all at 1:100). Appropriate isotype controls were used. Cells were gated as singlets > lymphocytes > CD4 + T cells or CD8 + T cells or B220 + B cells or Ly6G + Ly6C + neutrophils or Ly6C + CD11b + inflammatory monocytes or CD169 + CD11b + subcapsular macrophages or NK1.1 + NK cells or MHC-II + CD11c + CD11b + DCs or MHCII + CD11c + CD11b- DCs > pro-IL1β + or IFN-γ + (Supplementary Fig. 3B). All data was collected on Fortessa or LRSII flow cytometer (BD Biosciences) and the data analyzed using FlowJo (Tree Star).

### Serum endpoint titer ELISA

Peripheral blood was collect and subsequent centrifugation at 10,621 x *g* for 5 min to isolate the serum. Serum titers against ID93 were then evaluated by antibody capture ELISA. Briefly, Corning high bind 384 well plates (VWR International) were coated overnight at 4 °C with 2 µg/ml ID93 in coating buffer (eBioscience). Then, plates were blocked with 1% BSA-PBS and serum samples serially diluted. Detection antibodies were anti-mouse IgG2c (Southern Biotech Cat# 1079-05) or IgG1 HRP (Southern Biotech Cat# 1070-05). Plates were analyzed at 450 nm (ELx808, Bio-Tek Instruments Inc.) and endpoints were set as the minimum dilution at which values were lesser or equal to an OD of 0.2.

### Antigen binding

In order to assess the binding vs. non-binding question, we admixed stock concentration of a nanoalum formulation (0.6 mg/ml aluminum final) with a predetermined protein antigen amount (10 µg/ml). After incubating the protein-nanoalum mixture for a fixed temperature and time, the mixture was ultracentrifuged at 100,000 x *g* to ensure nanoalum sedimentation but not pelleting of the unbound protein. The supernatant was tested for the presence of protein using sodium dodecyl sulphate-polyacrylamide gel electrophoresis. The comparison of protein content in the supernatants of un-centrifuged and centrifuged samples provides an indirect assay to determine protein binding versus non-binding.

### Innate cytokine ELISAs

At indicated times following immunization dLNs were collected in PBS + 0.5% BSA + EDTA-free protease inhibitor cocktail (1:100; Sigma) + 10 µg/ml Brefeldin A (GolgiPlug, BD Biosciences) and manually homogenized. Supernatants were collected and total production of IL-18, IL-12p70 or IFN-γ was assessed using the corresponding Mouse Ready-Set-Go! ELISA kit (Affymetrix) according to the manufacturer’s instructions.

### Hemagglutination inhibition assays

Hemagglutination Inhibition (HAI) activity specific to A/PR/8/34 was performed as previously described in using 1% Turkey Red Blood Cells (TRBCs).^49^ Briefly, each serum sample was treated with receptor-destroying enzyme (RDE) overnight at 37 °C followed by heat inactivation to remove nonspecific inhibitors. HAI titer was determined by the reciprocal of the highest dilution of sera that completely inhibited the agglutination of TRBCs following addition of 4 HAU (hemagglutination units) of virus, starting at a 1:10 dilution and serially diluting twofold down a 96 V-bottom plate.

### Infection studies and viral titers

Mice were infected intranasally with 25 LD50 of influenza virus A/PR/8/34 (H1N1) 25 µL in phosphate-buffered saline (PBS). Mice were monitored for weight loss and other signs of virus-induced morbidity daily, such as ruffled fur and lethargy, and euthanized if weight loss exceeded 20% of initial body weight. Whole lungs of a subset of mice (*n* = 5 per group) were flash frozen on day 4 post infection for viral titer determination. Briefly, frozen lungs were ground using disposable tissue grinders in PBS, standardized based on weight and viral titers determined by 50% tissue culture infectious dose in Madin-Darby canine kidney (MDCK) cells using the Reed and Muench method.^50^

### Statistical analysis

All data are presented as mean ± SEM. Data were analyzed using GraphPad Prism 7 software (La Jolla, CA, USA) by one-way ANOVA (with Tukey’s multiple comparisons post-test), two-way ANOVA (with Bonferroni multiple comparisons post-test), or by unpaired Student *t*-tests as indicated for each experiment. Values were considered significantly different with *P* < 0.05 (*), *P* < 0.01 (**), *P* < 0.001 (***).

## Supplementary information

Supplementary Information

## Data Availability

The datasets generated during and/or analyzed during the current study are available from the corresponding author on reasonable request (MTO).

## Supplementary material

**Supplementary information** accompanies the paper on the *npj Vaccines* website (10.1038/s41541-018-0094-0).
