# Supplementary material for: Reprogramming the adjuvant properties of aluminum oxyhydroxide with nanoparticle technology
Source: NPJ Vaccines. 2019 Jan 3;4:1. doi: 10.1038/s41541-018-0094-0 (PMC6318334; doi:10.1038/s41541-018-0094-0)
Supplement: Supplementary file 1 — Supplementary Information [file 41541_2018_94_MOESM1_ESM.docx]

**Supplemental Figure 1: Microfluidized Alhydrogel is not size stable**

Alhydrogel was microfluidized for 10 passes at 30,000 psi and assessed for particle size changes over time.


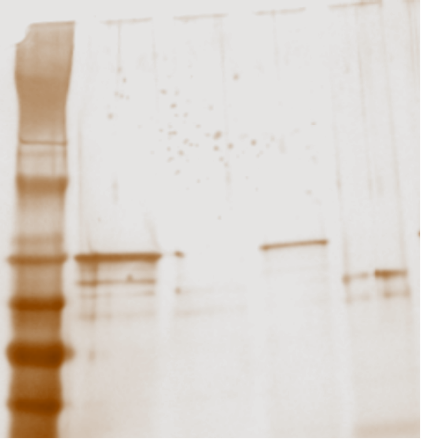


250 -

148 -

98 -

64 -

50 -

36 -

1 2 3 4 5

**Supplemental Figure 2: Full gel of Figure 4a**

ID93 was mixed with Alhydrogel, PAA, or PAA:nanoalum for detected by silver stain before and after centrifugation to pellet the alum particles. Lane 1: ladder in kDa with molecular weights indicated, 2: ID93, 3: ID93+Alhydrogel, 4: ID93+PAA:nanoalum 5: ID93+PEG:nanoalum.

**A**


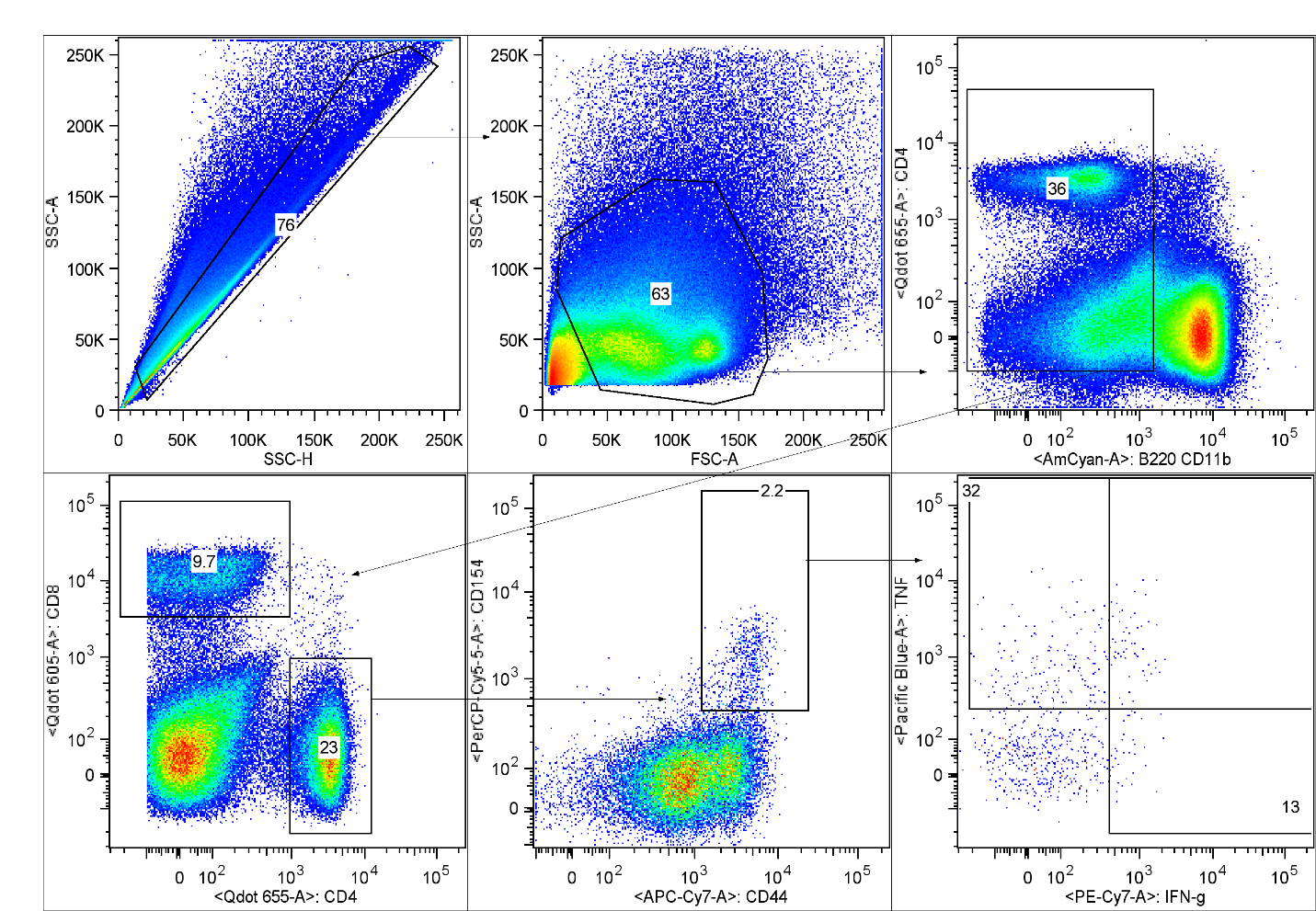


**B**


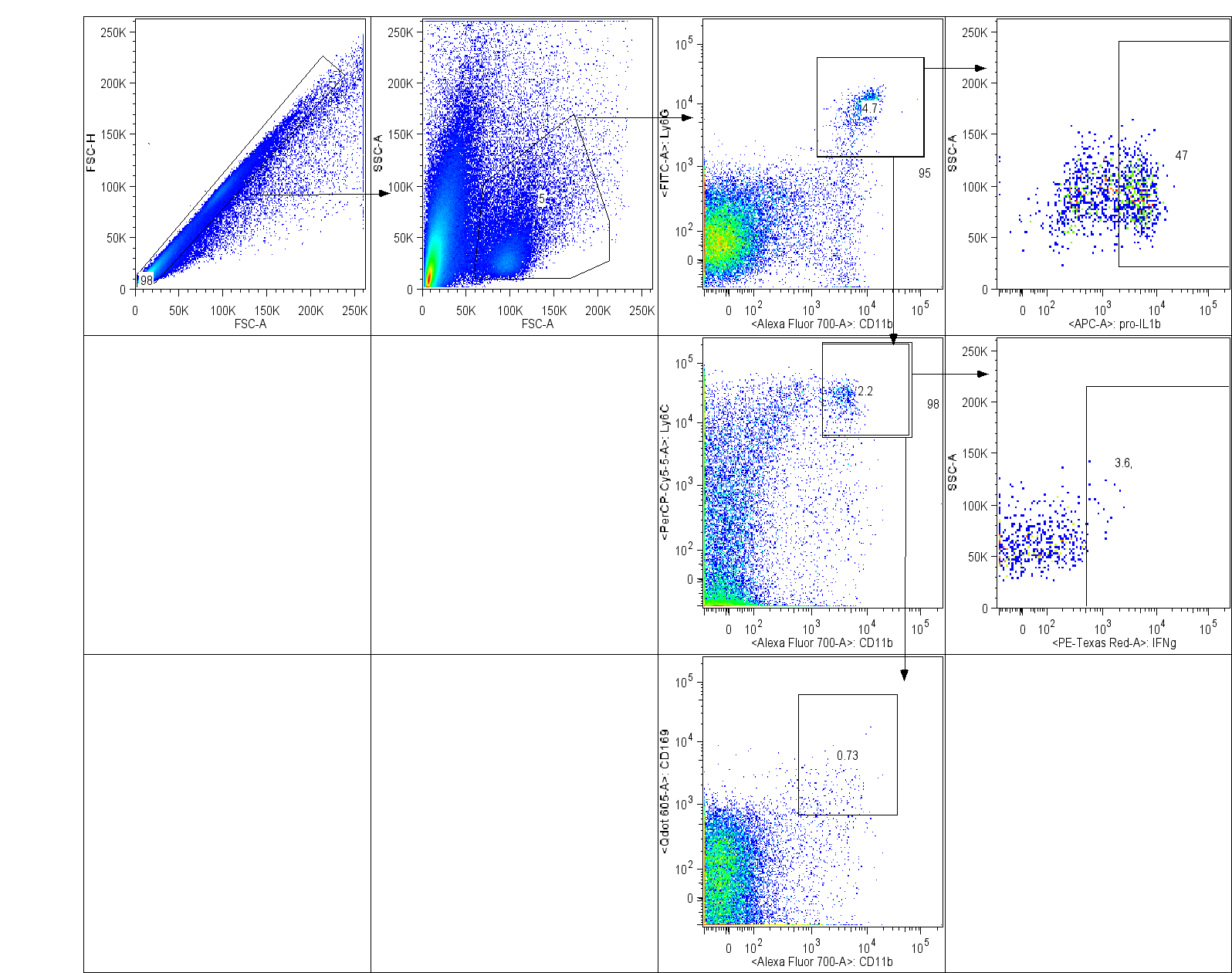


**Supplemental Figure 3:** Representative FACS gating strategies

(A) For the data in figures 3, 4b, and 5d cells were gated as singlets (SSC-A x SSC-H) then lymphocytes (SSC-A x FSC-A) then B220 and CD11b- CD8- CD4+ then CD4hi CD154+ then for IFN-γ and TNF expression. The frequency of CD154+ IFN-γ + or – TNF+ or - CD4 T cells were back calculated. (B) For the data in Figure 5b cells were gated as singlets (FSC-H x FSC-A), live cells (FSC-A x SSC-A), neutrophils were gated as CD11b+ Ly6G+ and analyzed for pro-IL-1β expression. Inflammatory monocytes (CD11b+ Ly6C+) were gated from the non-neutrophil population and analyzed for IFN-γ expression. Subcapsular macrophages were gated from the non-inflammatory monocyte population as CD11b+ CD169+.
